# Chemical properties and colors of fermenting materials in salmon fish sauce production

**DOI:** 10.1016/j.dib.2017.11.070

**Published:** 2017-11-27

**Authors:** Mitsutoshi Nakano, Yoshimasa Sagane, Ryosuke Koizumi, Yozo Nakazawa, Masao Yamazaki, Toshihiro Watanabe, Katsumi Takano, Hiroaki Sato

**Affiliations:** a Department of Food and Cosmetic Science, Faculty of Bioindustry, Tokyo University of Agriculture, 196 Yasaka, Abashiri Hokkaido 099-2493, Japan; b The Organization for the Promotion of International Relationship, 709, 1-1-7 Motoakasaka Minato-ku, Tokyo 107-0051, Japan; c Department of Applied Biology and Chemistry, Faculty of Applied Bioscience, Tokyo University of Agriculture, 1-1-1 Sakuragaoka, Setagaya-ku, Tokyo 156-8502, Japan

**Keywords:** Fish sauce, Chum salmon, Fermentation, Chemical properties, Color

## Abstract

This data article reports the chemical properties (moisture, pH, salinity, and soluble solid content) and colors of fermenting materials in salmon fish sauce products. The fish sauce was produced by mixing salt with differing proportions of raw salmon materials and fermenting for three months; the salmon materials comprised flesh, viscera, an inedible portion, and soft roe. Chemical properties and colors of the unrefined fish sauce (*moromi*), and the refined fish sauce, were analyzed at one, two, and three months following the start of fermentation. Data determined for all products are provided in table format.

**Specifications Table**

**Table:** 

Subject area	*Agricultural science*
More specific subject area	*Food chemistry*
Type of data	*Table*
How data were acquired	Chemical properties of fish sauce products were measured using a moisture analyzer (MX-50 A&D, Japan), salt meter (B-721, HORIBA, Japan), and pH meter (D-52, HORIBA). Colors of products were analyzed using a color difference meter (CR-400, Minolta, Japan).
Data format	*Raw, analyzed*
Experimental factors	*Fish sauce was produced from raw salmon materials, which were purchased at a local market.*
Experimental features	Chemical properties and colors of fermenting salmon fish sauce. Colors as represented by L*, a*, and b* values.
Data source location	*Abashiri, Hokkaido, Japan*
Data accessibility	All data are presented in this article.

**Value of the data**

• The data would be available as reference for culinary studies on fish sauces and related products.
• The data will be useful for nutritional assessments of fish sauce products based on their chemical properties.
• The data will be useful for discussions on the metabolism of fish sauce chemical components during fermentation.

## 1 Data

This article presents data on moisture, pH, salinity, and soluble solid content of unrefined fish sauce, (*moromi*; Table 1) and filtered fish sauce (Table 2) throughout the fermentation process; we also report colorimetric data (Table 3 for *moromi*; Table 4 for fish sauce). Japanese terms in this article are explained in Table S1.

**Table 1:** Chemical properties of *moromi* products in the fish sauce fermentation process.

	Fermentation period (months)	Product ID									
		A	B	C	D	E	F	G	H	I	J
Moisture (%)	0	60.47	62.60	57.09	58.31	56.67	56.81	55.37	57.65	58.32	58.03
	1	55.72	59.50	57.52	59.32	54.43	56.37	54.94	56.92	55.71	57.65
	2	57.20	57.69	59.19	59.43	51.24	53.18	54.76	56.51	53.47	57.50
	3	60.21	59.55	62.15	59.66	49.69	51.36	51.54	54.01	55.50	50.87
pH	0	5.13	5.42	5.05	5.19	5.45	5.62	5.53	5.56	5.77	5.36
	1	5.74	5.53	5.78	5.47	5.61	5.48	5.71	5.49	5.83	5.4
	2	5.67	5.5	5.72	5.44	5.47	5.45	5.52	5.37	5.78	5.3
	3	5.4	5.43	5.54	5.23	5.53	5.16	5.39	5	5.51	4.99
Salinity (%)	0	22.00	21.00	24.67	22.67	24.00	19.00	23.00	21.00	22.67	20.67
	1	24.33	20.33	23.00	19.67	24.33	19.00	23.67	20.00	24.33	21.00
	2	22.00	20.67	22.33	20.33	23.00	20.33	23.67	19.00	23.33	18.33
	3	21.67	20.33	22.33	20.00	22.67	20.00	23.33	19.33	23.00	18.00
Soluble solid content (%)	0	39.53	37.40	42.91	41.69	43.33	43.19	44.63	42.35	41.68	41.97
	1	44.28	40.51	42.48	40.69	45.58	43.64	45.06	43.08	44.30	42.35
	2	42.80	42.31	40.82	40.58	48.76	46.82	45.24	43.49	46.53	42.51
	3	39.79	40.45	37.85	40.34	50.31	48.64	48.46	45.99	44.50	49.13
Unsalted soluble solid content (%)	0	17.53	16.40	18.24	19.02	19.33	24.19	21.63	21.35	19.01	21.30
	1	19.95	20.18	19.48	21.02	21.25	24.64	21.39	23.08	19.97	21.35
	2	20.80	21.64	18.49	20.25	25.76	26.49	21.57	24.49	23.20	24.18
	3	18.12	20.12	15.52	20.34	27.64	28.64	25.13	26.66	21.50	31.13

**Table 2:** Chemical properties of fish sauce products in the fish sauce fermentation process.

	Fermentation period (months)	Product ID									
		A	B	C	D	E	F	G	H	I	J
Moisture (%)	1	68.44	66.51	n.d.	n.d.	67.95	65.02	65.02	63.35	n.d.	n.d.
	2	67.65	64.86	65.71	60.91	66.40	64.35	65.60	63.35	n.d.	n.d.
	3	67.63	64.89	63.80	63.59	66.98	62.86	64.81	62.31	60.46	58.91
pH	1	5.72	5.53	n.d.	n.d.	5.67	5.50	5.51	5.43	n.d.	n.d.
	2	5.67	5.27	5.65	5.36	5.52	5.31	5.55	5.30	n.d.	n.d.
	3	5.36	5.29	5.58	5.33	5.45	5.35	5.56	5.20	5.70	5.17
Salinity (%)	1	29.00	27.00	n.d.	n.d.	29.33	27.00	26.67	25.00	n.d.	n.d.
	2	28.67	27.00	25.00	23.00	28.00	26.00	28.00	26.00	n.d.	n.d.
	3	28.00	26.67	24.33	22.67	27.00	25.67	27.00	25.33	26.33	25.33
Soluble solid content (%)	1	31.56	33.49	n.d.	n.d.	32.05	34.98	34.98	36.65	n.d.	n.d.
	2	32.35	35.14	34.29	39.09	33.60	35.65	34.40	36.65	n.d.	n.d.
	3	32.37	35.11	36.20	36.41	33.02	37.14	35.19	37.69	39.54	41.09
Unsalted soluble solid content (%)	1	2.56	6.49	n.d.	n.d.	2.72	7.98	8.31	11.65	n.d.	n.d.
	2	3.68	8.14	9.29	16.09	5.60	9.65	6.40	10.65	n.d.	n.d.
	3	4.37	8.44	11.87	13.74	6.02	11.47	8.19	12.36	13.21	15.76

n.d.: No data; filtrate was not available because of liquid component shortage.

**Table 3:** Colors of *moromi* products in the fish sauce fermentation process.

Lab color space	Fermentation period (months)	Product ID									
		A	B	C	D	E	F	G	H	I	J
L* (lightness)	0	53.80	49.16	49.31	63.00	55.76	52.43	54.41	55.44	80.53	76.40
	1	57.29	51.68	58.79	59.77	57.51	57.24	60.51	51.30	49.57	65.25
	2	53.95	58.69	61.75	48.16	45.95	43.67	53.69	45.93	68.25	61.92
	3	48.63	40.66	47.84	52.88	63.79	54.94	54.92	41.72	70.37	58.44
a* (red-green)	0	6.14	8.94	10.99	8.79	7.77	16.26	11.05	11.77	3.04	3.29
	1	5.26	7.70	5.80	5.80	5.40	9.78	7.87	9.68	12.19	5.18
	2	4.49	7.80	7.90	8.13	6.36	7.84	7.84	8.09	4.34	1.50
	3	5.62	4.40	5.83	6.12	12.29	12.13	8.46	7.40	2.54	13.32
b* (yellow-blue)	0	16.42	18.61	22.35	20.95	19.66	27.07	20.57	19.41	19.36	19.78
	1	20.68	24.17	23.45	23.45	26.28	27.76	24.64	23.99	42.90	29.94
	2	20.57	25.01	25.36	24.63	21.82	22.50	23.74	25.09	30.17	7.48
	3	20.15	11.94	17.43	12.91	34.34	22.85	20.80	11.43	22.50	32.07

**Table 4:** Colors of fish sauce products in the fish sauce fermentation process.

Lab color space	Fermentation period (months)	Product ID									
		A	B	C	D	E	F	G	H	I	J
L* (lightness)	1	59.70	56.63	n.d.	n.d.	60.27	53.92	72.26	79.15	n.d.	n.d.
	2	57.07	59.70	73.59	67.27	60.91	56.38	69.78	52.17	n.d.	n.d.
	3	57.05	51.95	65.61	57.08	65.88	32.25	55.06	35.59	66.79	36.39
a* (red-green)	1	−0.19	0.19	n.d.	n.d.	−1.04	1.06	−1.98	1.55	n.d.	n.d.
	2	−1.23	1.54	−1.79	3.80	−0.68	7.67	−1.68	14.74	n.d.	n.d.
	3	−0.15	12.95	−1.80	17.19	1.62	25.73	5.95	27.10	−3.12	20.93
b* (yellow-blue)	1	5.50	9.48	n.d.	n.d.	15.80	12.97	23.42	44.01	n.d.	n.d.
	2	15.82	39.64	15.14	42.53	32.39	55.25	36.57	62.55	n.d.	n.d.
	3	31.87	60.81	33.28	72.32	48.84	32.07	56.71	57.04	19.33	33.12

n.d.: No data; filtrate was not available because of liquid component shortage.

## 2 Experimental design, materials, and methods

### 2.1 Design

We used female ‘*Bunasake’* and ‘*Ginke’* chum salmon (*Oncorhynchus keta*) from Hokkaido, Japan. *‘Bunasake*’, which is chum salmon after the egg-laying period, has a low market value, because these fish abstain from eating during their pre-spawning run upstream, and the fat in their flesh is depleted. On the other hand, ‘*Ginke’*, which is chum salmon before the period of migration and spawning, has high market value, because the flesh of these fish is relatively high in fat content. We also used *shio-koji*, a salt-marinated rice malt, as the fermentation starter. For the color measurement, the L* value that represents brightness, a* value that represents red/green and b* that represents yellow/blue were employed to assess lightness and degree of color shift of the products, respectively.

### 2.2 Materials

The ‘*Bunasake’* salmon and ‘*Ginke’* salmon were purchased at a local market in Abashiri, Hokkaido. *Shio-koji* containing 12.3% salt (Kurashige jozo Co. Ltd., Abashiri Japan) and salt (Shokuen, The Salt Industry Center of Japan, Tokyo, Japan) were used for fish sauce production.

### 2.3 Fish sauce production

Raw salmon was dissected into flesh, viscera, an inedible portion, and soft roe. Each portion was minced using a food processor. Each minced portion and salt were mixed at various proportions (Table S2), and left for three months in a 37 °C constant temperature incubator (DG-82, Yamato, Japan). The chemical properties and colors of the *moromi* (unrefined fish sauce) and filtered fish sauce were measured at one, two, and three months following the start of fermentation.

### 2.4 Physicochemical analyses

Salinity and pH were measured using a salt meter (B-721; HORIBA, Japan) and a pH meter (D-52; HORIBA). Water content was measured according to the methods published by the Association of Official Analytical Chemists [1], [2]. Soluble solid content (%) was defined as content excluding the water content. Unsalted soluble solid content (%) was defined as the soluble solid content excluding the salt content.

### 2.5 Color analysis

The colors of dried salmon products were measured using a colorimeter (CR-400; Konica Minolta, Tokyo, Japan). The measured data were expressed in Lab color space in which the tree dimensions L* for lightness, and a* and b* for the color opponents green/red and blue/yellow.
