# Supplementary material for: Chemical properties and colors of fermenting materials in salmon fish sauce production
Source: Data Brief. 2017 Nov 27;16:483–8. doi: 10.1016/j.dib.2017.11.070 (PMC5725210; doi:10.1016/j.dib.2017.11.070)
Supplement: Supplementary file 2 — Supplementary material [file mmc2.docx]

**Table S1.** Glossary of Japanese terms in this article

| Japanese term | Meaning |
| --- | --- |
| *moromi* | Unrefined fish sauce. The filtrate of the *moromi* is fish sauce. |
| ‘Bunasake’ | Chum salmon after egg-laying period |
| ‘Ginke’ | Chum salmon before laying period |
| shio-koji | A salt-marinated rice malt |

**Table S2.** Proportions (%) of the materials used in fish sauce production.

| Product ID | Fish material | Flesh | Viscera | Inedible portion* | Soft roe | Salt | *"Shio-koji"* | Total |
| --- | --- | --- | --- | --- | --- | --- | --- | --- |
| A | ‘*Bunasake’* | 55.0 | 5.0 | 15.0 | - | 25.0 | - | 100 |
| B |  | 49.5 | 4.5 | 13.5 | - | 22.5 | 10.0 | 100 |
| C |  | 35.0 | 5.0 | 15.0 | 20.0 | 25.0 | - | 100 |
| D |  | 31.5 | 4.5 | 13.5 | 18.0 | 22.5 | 10.0 | 100 |
| E | ‘*Ginke’* | 55.0 | 5.0 | 15.0 | - | 25.0 | - | 100 |
| F |  | 49.5 | 4.5 | 13.5 | - | 22.5 | 10.0 | 100 |
| G |  | 35.0 | 5.0 | 15.0 | 20.0 | 25.0 | - | 100 |
| H |  | 31.5 | 4.5 | 13.5 | 18.0 | 22.5 | 10.0 | 100 |
| I | Soft roe | - | - | - | 75.0 | 25.0 | - | 100 |
| J |  | - | - | - | 67.5 | 22.5 | 10.0 | 100 |

*Inedible portion includes fish heads, backbones, and fins.
